# Supplementary material for: Construction, Physical Properties and Foaming Behavior of High-Content Lignin Reinforced Low-Density Polyethylene Biocomposites
Source: Polymers (Basel). 2022 Jun 30;14(13):2688. doi: 10.3390/polym14132688 (PMC9269087; doi:10.3390/polym14132688)
Supplement: Supplementary file 1 [file polymers-14-02688-s001.zip › polymers-1755649-supplementary.pdf]

polymers

MDPI

Supplementary Materials: Construction, Physical Properties and Foaming Behavior of High-content Lignin Reinforced Low-density Polyethylene Biocomposites

Seo-Hwa Hong and Seok-Ho Hwang

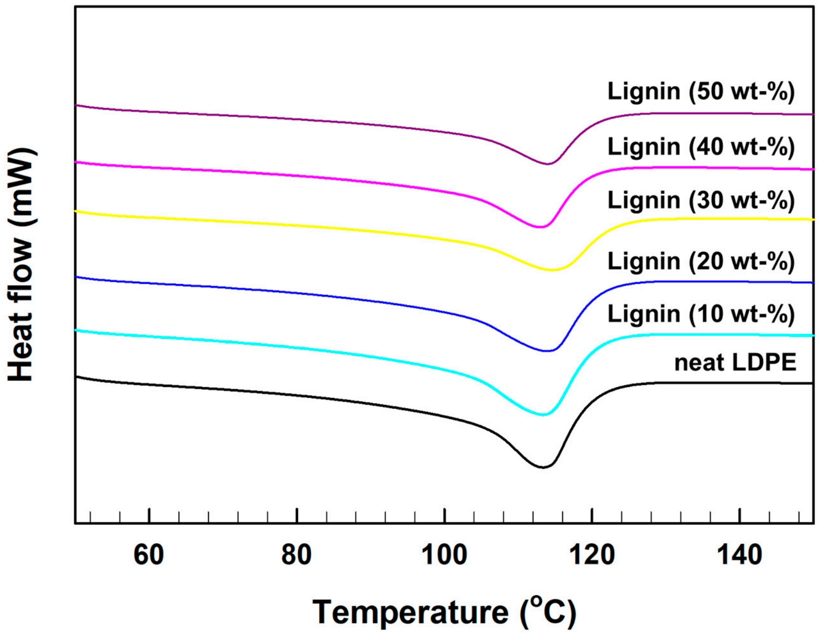

A

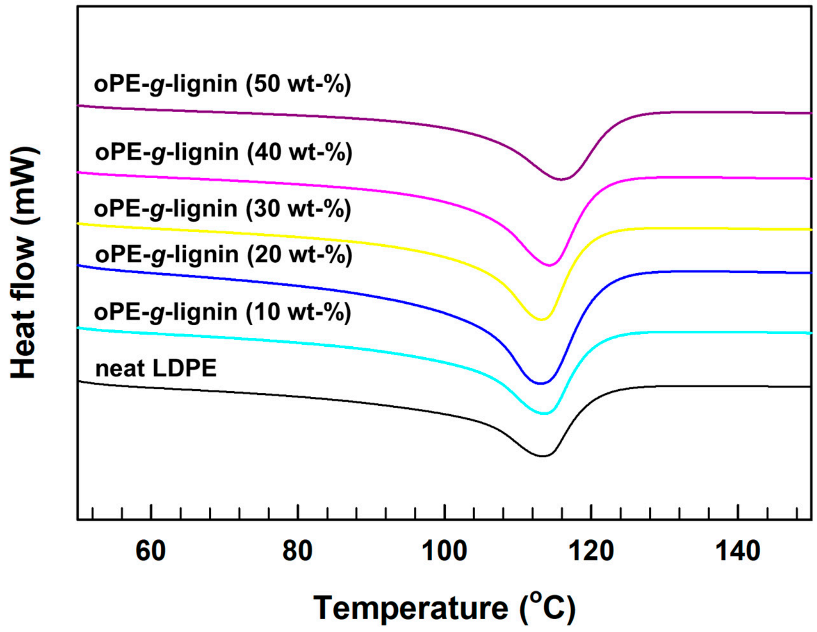

B

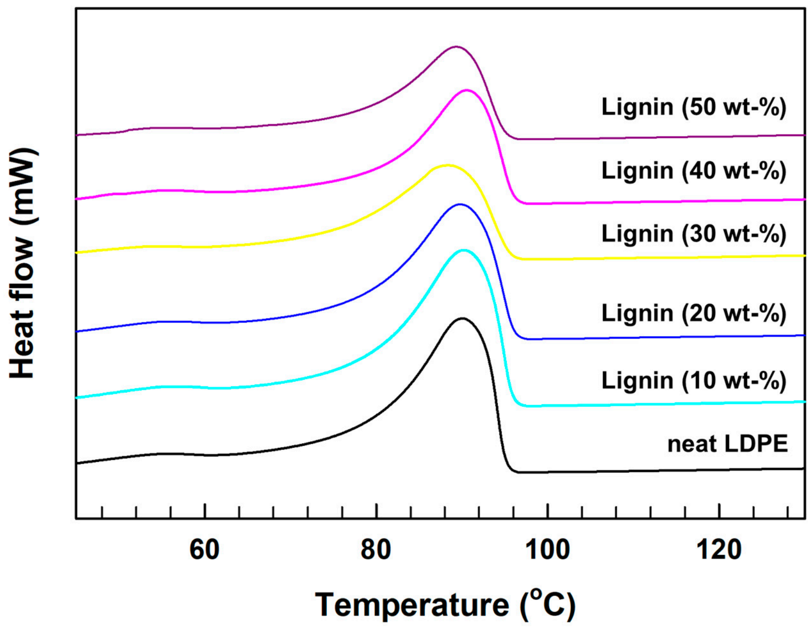

C

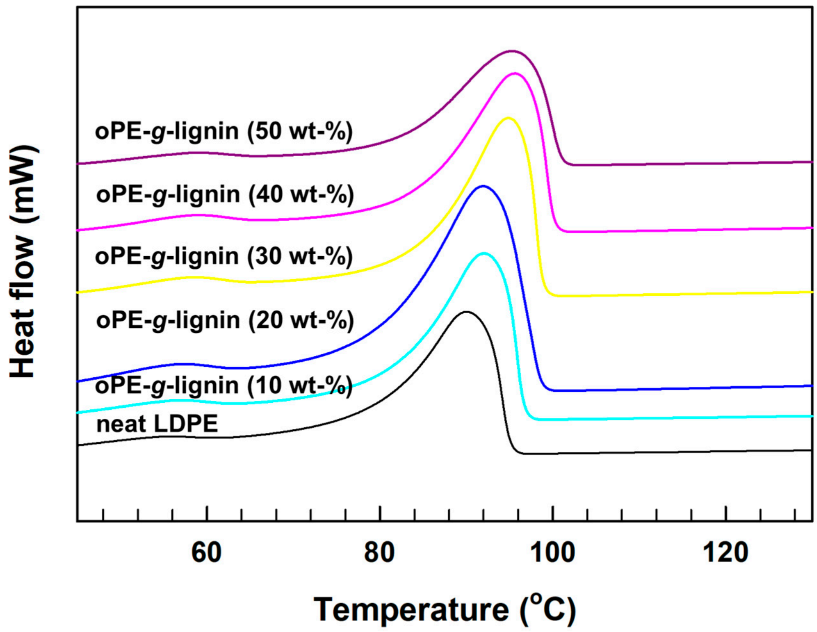

D

Figure S1. DSC thermograms: (A) heating curve for pristine lignin/LDPE biocomposites, (B) heating curve for oPE-*g*-lignin/LDPE biocomposite, (C) cooling curve for pristine lignin/LDPE biocomposites, (D) cooling curve for oPE-*g*-lignin/LDPE biocomposite.

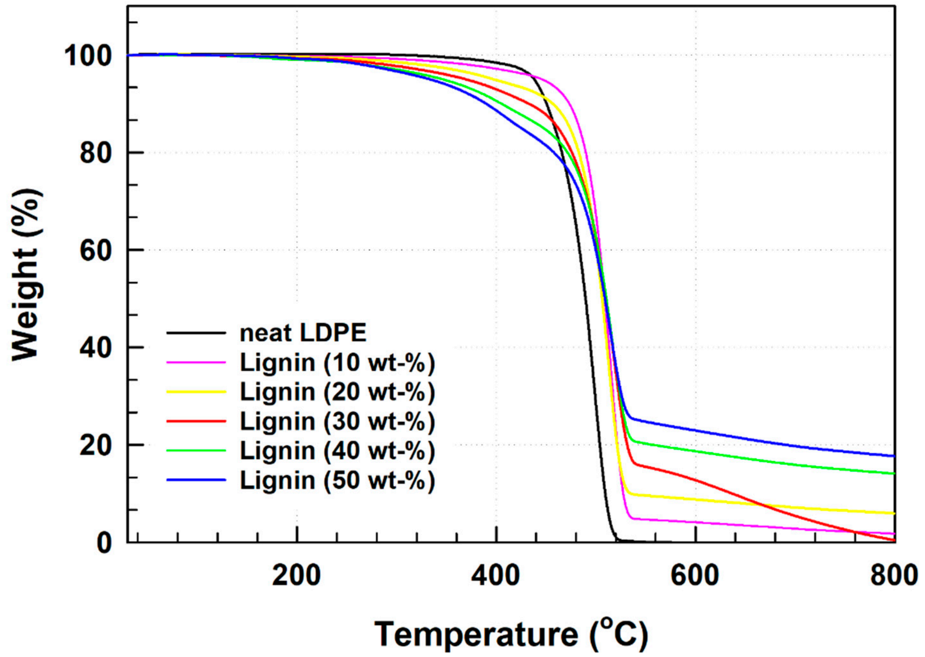

A

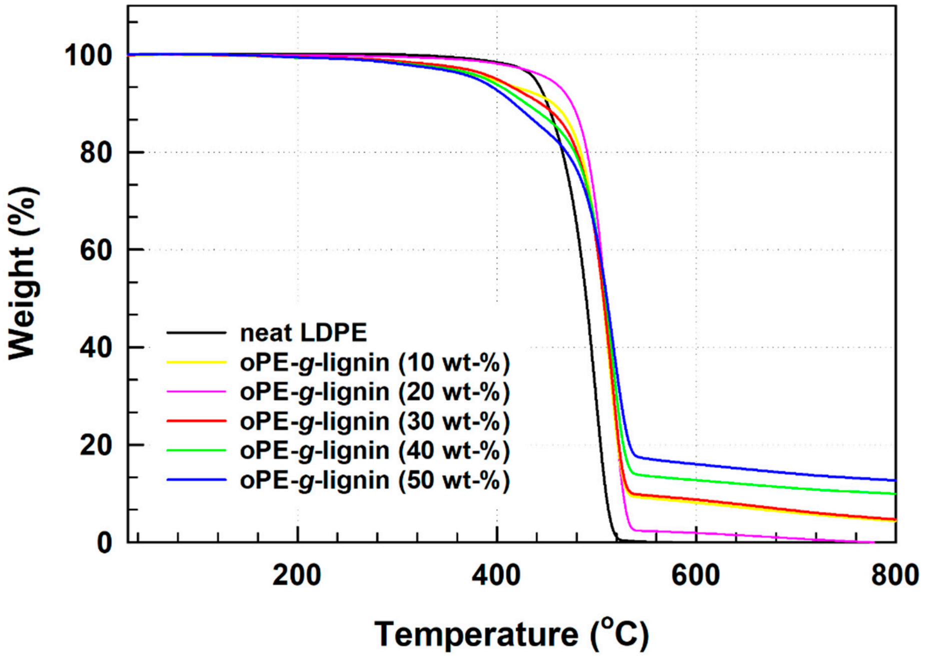

B

Figure S2. TGA thermograms for pristine lignin/LDPE biocomposites (A) and oPE-*g*-lignin/LDPE biocomposites (B).

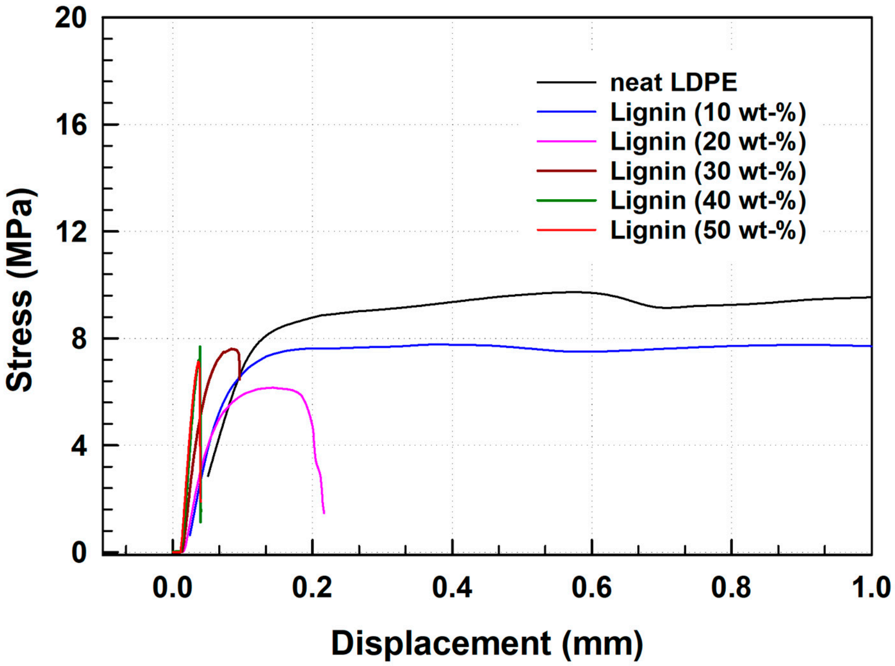

A

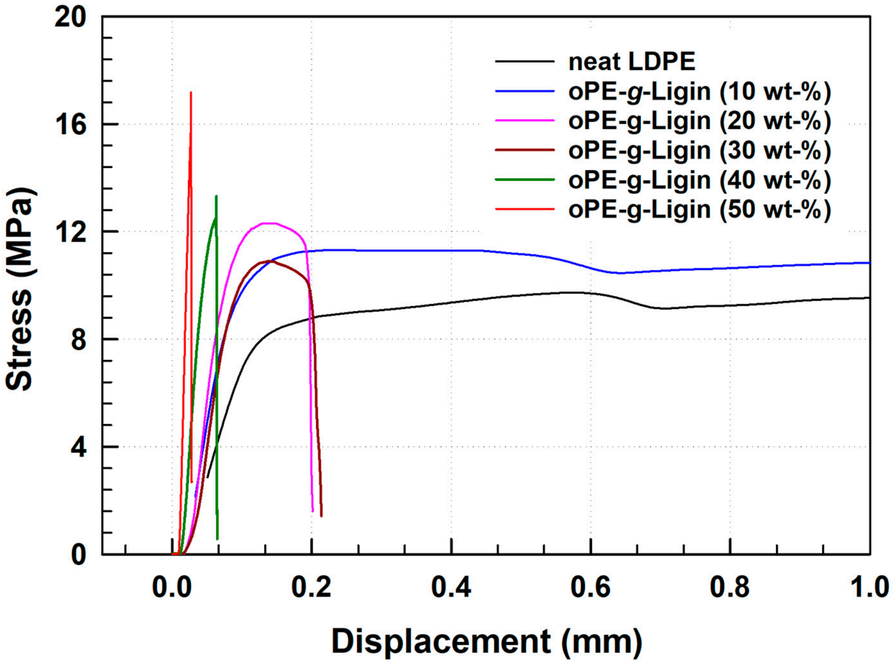

B

Figure S3. Stress-strain curves for pristine lignin/LDPE biocomposites (A) and oPE-*g*-lignin/LDPE biocomposites (B).
